# Supplementary material for: Functional neuronal circuitry and oscillatory dynamics in human brain organoids
Source: Nat Commun. 2022 Jul 29;13:4403. doi: 10.1038/s41467-022-32115-4 (PMC9338020; doi:10.1038/s41467-022-32115-4)
Supplement: Supplementary file 3 — Description of Additional Supplementary Files [file 41467_2022_32115_MOESM3_ESM.pdf]

Description of Additional Supplementary Files

File Name: Supplementary Data 1.
Description: Organoid age when the onset of synchronized neuronal population bursts occur are listed in a table for six organoids.
