# Supplementary material for: Functional neuronal circuitry and oscillatory dynamics in human brain organoids
Source: Nat Commun. 2022 Jul 29;13:4403. doi: 10.1038/s41467-022-32115-4 (PMC9338020; doi:10.1038/s41467-022-32115-4)
Supplement: Supplementary file 5 — Reporting Summary [file 41467_2022_32115_MOESM5_ESM.pdf]

nature research

Corresponding author(s): Kenneth S. Kosik
Tal Sharf

Last updated by author(s): Jun 1, 2022

Reporting Summary

Nature Research wishes to improve the reproducibility of the work that we publish. This form provides structure for consistency and transparency in reporting. For further information on Nature Research policies, see our Editorial Policies and the Editorial Policy Checklist.

Statistics

For all statistical analyses, confirm that the following items are present in the figure legend, table legend, main text, or Methods section.

n/a	Confirmed	
☐	☒	The exact sample size (n) for each experimental group/condition, given as a discrete number and unit of measurement
☐	☒	A statement on whether measurements were taken from distinct samples or whether the same sample was measured repeatedly
☐	☒	The statistical test(s) used AND whether they are one- or two-sided<br>*Only common tests should be described solely by name; describe more complex techniques in the Methods section.*
☐	☒	A description of all covariates tested
☐	☒	A description of any assumptions or corrections, such as tests of normality and adjustment for multiple comparisons
☐	☒	A full description of the statistical parameters including central tendency (e.g. means) or other basic estimates (e.g. regression coefficient) AND variation (e.g. standard deviation) or associated estimates of uncertainty (e.g. confidence intervals)
☐	☒	For null hypothesis testing, the test statistic (e.g. F, t, r) with confidence intervals, effect sizes, degrees of freedom and P value noted<br>*Give P values as exact values whenever suitable.*
☒	☐	For Bayesian analysis, information on the choice of priors and Markov chain Monte Carlo settings
☒	☐	For hierarchical and complex designs, identification of the appropriate level for tests and full reporting of outcomes
☒	☐	Estimates of effect sizes (e.g. Cohen's d, Pearson's r), indicating how they were calculated

Our web collection on statistics for biologists contains articles on many of the points above.

Software and code

Policy information about availability of computer code

Data collection	Maxlab Live, Maxwell Biosystems; SpikeGLX, Bill Karsh at Janelia Research Campus; Olympus Fluoview software version 4.2; Illumina Nextseq500 platform
Data analysis	MATLAB (MathWorks), Python, R; SpikeInterface 0.13.0 in Python 3.6 (https://github.com/SpikeInterface); MATLAB (version 2018b) code for MEA analysis as described in methods utilizing previously published code in C, can be found here https://github.com/CCutts/Detecting_pairwise_correlations_in_spike_trains, and has been adapted to MATLAB which can be found here https://github.com/Timothysit/organoids; Custom code for the visualization of organoid network activity is available at https://github.com/KosikOrganoid/Intrinsic-activity-code; STAR in Drop-seq tools package version 1.13 (https://github.com/broadinstitute/Drop-seq/releases); Seurat 3.0 in R version 3.6; DoubletFinder 2.0.3 in R version 3.6.

For manuscripts utilizing custom algorithms or software that are central to the research but not yet described in published literature, software must be made available to editors and reviewers. We strongly encourage code deposition in a community repository (e.g. GitHub). See the Nature Research guidelines for submitting code & software for further information.

Data

Policy information about availability of data

All manuscripts must include a data availability statement. This statement should provide the following information, where applicable:

- Accession codes, unique identifiers, or web links for publicly available datasets
- A list of figures that have associated raw data
- A description of any restrictions on data availability

The data support the findings of this study are available within the article and its supplementary information. Electrophysiology recordings can be found here https://doi.org/10.25349/D9031Z. Source data for ssRNA-seq are provided with the paper.

Field-specific reporting

Please select the one below that is the best fit for your research. If you are not sure, read the appropriate sections before making your selection.

- [x] Life sciences
- [ ] Behavioural & social sciences
- [ ] Ecological, evolutionary & environmental sciences

For a reference copy of the document with all sections, see nature.com/documents/nr-reporting-summary-flat.pdf

Life sciences study design

All studies must disclose on these points even when the disclosure is negative.

Sample size	Sample size was determined by the number of oganoid samples available.
Data exclusions	Due to intrinsic neurophysiological variability occurring during brain organoid development, organoids exhibited a wide range in the number of active spiking units. Organoids exhibiting significantly smaller proportion of spiking units were omitted from some portions of the network analysis. The sample size and excluded sets are mention in the text and figure legends.
Replication	Extracellular recordings were performed on six organoids positioned on 2D CMOS arrays and pharmacological manipulations were performed on a subset (n = 4) of that cohort. Additional recordings were performed on three whole organoids using CMOS shank electrodes. Single cell rna-seq and histology was performed on three organoids to confirm the presence of cell types.
Randomization	Spike time matrices were randomized to determine a lower bound for functional connectivity and feature significance as detailed in the Methods. Pharmacological manipulations were performed on available organoid slices.
Blinding	Data analysis was not blinded. Computational algorithms were applied uniformly across all data sets analyzed without assumption of a trend. Blinding during data collection was not possible because organoids used in this study followed identical generation protocols and were derived from the same cell line. Pharmacological manipulations were administered manually and could not be carried out without knowledge of drug concentration. All organoids available for pharmacological experiments were performed simultaneously.

Reporting for specific materials, systems and methods

We require information from authors about some types of materials, experimental systems and methods used in many studies. Here, indicate whether each material, system or method listed is relevant to your study. If you are not sure if a list item applies to your research, read the appropriate section before selecting a response.

Materials & experimental systems

n/a	Involved in the study	
[ ]	[x]	Antibodies
[ ]	[x]	Eukaryotic cell lines
[x]	[ ]	Palaeontology and archaeology
[x]	[ ]	Animals and other organisms
[x]	[ ]	Human research participants
[x]	[ ]	Clinical data
[x]	[ ]	Dual use research of concern

Methods

n/a	Involved in the study	
[x]	[ ]	ChIP-seq
[x]	[ ]	Flow cytometry
[x]	[ ]	MRI-based neuroimaging

Antibodies

Antibodies used	Primary antibodies with their corresponding dilutions were:anti-GFAP (abcam; ab53554; goat 1:500), anti-connexin 43 (Millipore; MAB3067, mouse 1:200), anti-MAP2 (GeneTex; GTX82661; chicken 1:500), anti-SMI312 (BioLegend; 801701; mouse 1:500), anti-Parvalbumin (abcam; ab11427; rabbit 1:200), anti-GAD65 (GeneTex; GTX113192; rabbit 1:200), anti-synaptobrevin (Synaptic Systems; 104-211; mouse 1:500)
Validation	All antibodies used were commercially purchased and were validated for their respective application by their manufacturer.

Eukaryotic cell lines

Policy information about cell lines

Cell line source(s)	Human induced pluripotent stem cells (iPSCs)
Authentication	Human iPSCs were characterized using standard methods, analyzed for pluripotency markers and chromosomal abnormalities by G-band karyotyping and were confirmed to possess the appropriate genotype by Sanger sequencing.

Mycoplasma contamination	Mycoplasma testing was routinely performed on organoids and iPSCs and all cells tested negative
Commonly misidentified lines (See ICLAC register)	No commonly misidentified cell lines were used.
